# Ocular features in mucopolysaccharidosis: diagnosis and treatment

**DOI:** 10.1186/s13052-018-0559-9

**Published:** 2018-11-16

**Authors:** Alessandra Del Longo, Elena Piozzi, Fiammetta Schweizer

**Affiliations:** 1 Pediatric Department, ASST Grande Ospedale Metropolitano Niguarda, Piazza Ospedale Maggiore 3, 20162 Milan, Italy; 2 Department of Ophthalmology, Ospedale San Gerardo, Via G. B. Pergolesi 33, 20052 Monza, MB Italy

**Keywords:** Eye manifestations, Mucopolysaccharidosis, MPS, Corneal clouding, Diagnosis MPS, Treatment MPS

## Abstract

Mucopolysaccharidoses (MPS) are a group of rare lysosomal storage disorders characterized by the accumulation of glycosaminoglycans (GAGs) in different parts of the eye. Ocular problems are very common in MPS children, and the cornea, sclera, trabecular meshwork, retina, and optic nerve may all be involved. Early diagnosis is very important to preserve the visual function, and the diagnosis requires experience and different evaluations. Follow-up is mandatory to allow a correct pathway to consequent therapy. This article aims to provide a review of ocular alterations and treatment options in MPS. The ophthalmologist is sometimes the first physician who can suspect a metabolic disease and can help to make the correct diagnosis. It is important to stimulate awareness of MPS among ophthalmologists.

## Background

Ocular problems in mucopolysaccharidoses (MPS) are very common, with different presentations in terms of time of onset and severity [1–5]. Glycosaminoglycan (GAG) accumulation is the first cause of ocular manifestations. Eye alterations may involve the sclera, cornea, trabecular meshwork, retina, optic nerve, and posterior visual pathways [2, 3]. Amblyopia, strabismus, and large refraction defects are frequent and need adequate correction for better visual development [3]. Early diagnosis is very important to improve both the management and outcome [6].

Diagnostic delays are not uncommon in patients with MPS. The ophthalmologist plays a crucial role in early diagnosis and follow-up. The ocular manifestations are present in all types of MPS, but are particularly common in MPS I, VI, and VII (Table 1).

**Table 1:** Overview of ocular features in mucopolysaccharidoses (MPS)

	Ocular manifestations				
MPS type	Corneal clouding	Retinopathy	Glaucoma	Optic nerve abnormalities	Other
MPS I-S (Scheie)	+	++	+	+	
MPS I-HS (Hurler-Scheie)	++	++	++	++	
MPS I-H (Hurler)	+++	+ +	++	+	
MPS II (Hunter)	+	+ +	+	++	
MPS III (Sanfilippo)	+	+ + +	+	+	Bushy eyebrows, late blindness
MPS IV (Morquio)	+	+ +	+	+	Pseudoexophthalmos
MPS VI (Maroteaux-Lamy)	+++	Unknown	++	++	
MPS VII (Sly)	++	Unknown	++	++	Colobomas of the iris
MPS IX (Natowicz)	Unknown	Unknown	Unknown	Unknown	

Modified from [25]

+ mild, ++ moderate, +++ severe

Even in attenuated phenotypes of MPS I, corneal clouding is found in approximately 70% of patients at a median age of 4 years in MPS I Hurler/Scheie (H/S) and 10 years in MPS IS [7].

### Ocular manifestations in MPS

#### Corneal clouding

Corneal clouding is caused by an accumulation of granules of a yellowish-grey colour made up of GAGs that are deposited in all corneal layers and by a displacement of collagen fibrils in the stroma. Excessive GAG storage in the cornea affects keratocyte size and disrupts the regular network of collagen fibrils in the stroma causing corneal clouding [2].

**Fig. 1:**
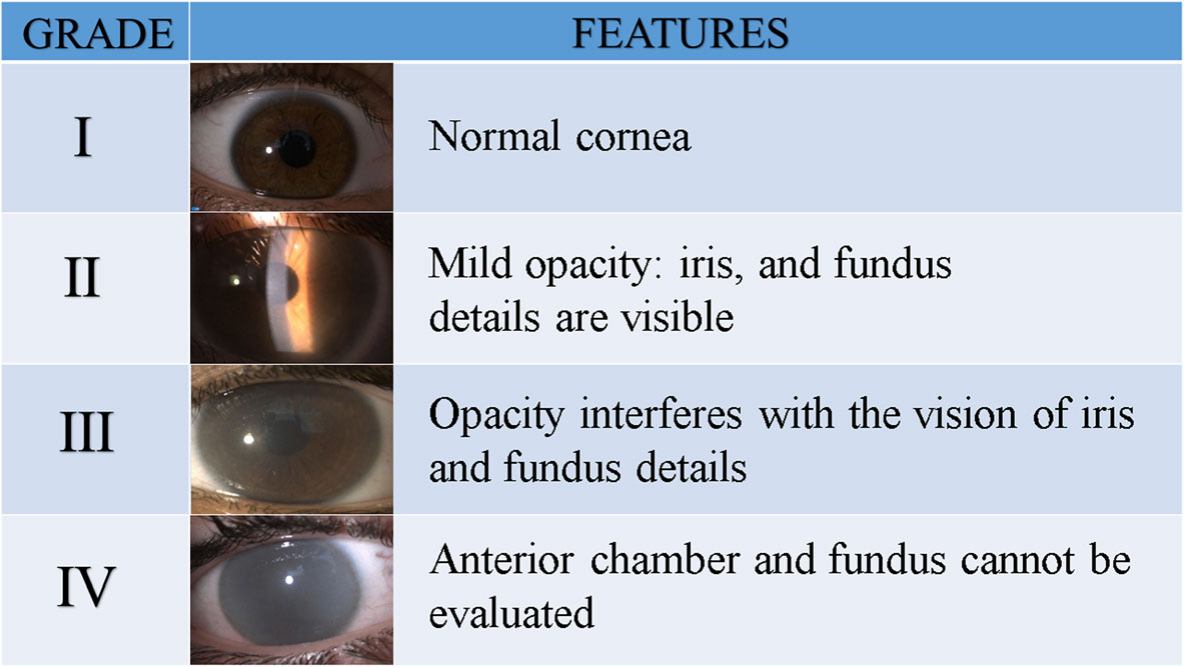
Classification of corneal clouding

Corneal clouding is diffuse and slowly progressive with a consequent loss of visual acuity (Fig. 1). It affects the whole cornea from limbus to limbus and degenerations from an anatomopathological point of view are also present in the conjunctiva and sclera [3, 4]. Corneal opacity, if dense, does not allow the examination of the lens and posterior segment (vitreous and retina). According to the literature, corneal clouding is more frequent in MPS 1H, as reported in Table 2.

**Table 2:** Corneal severity clouding in the patients with different types of mucopolysaccharidoses (MPS) evaluated in our department

	Corneal clouding			Corneal layer involved		
MPS type	+++	++	+	Stroma	Endothelium	Epithelium
I (*n* = 17)	12	3		15		
II (*n* = 14)		1	3	3		1
IIIA (*n* = 3)						
IIIB (*n* = 3)						
IV (*n* = 10)	3	3	2	6	1	1
VI (*n* = 4)	2	2		4		2

+ mild, ++ moderate, +++ severe

Despite systemic enzyme replacement therapy (ERT), clearing of the cornea is unusual, especially if the therapy is initiated relatively late. Most reports suggest a stabilizing impact of ERT on corneal clouding and visual acuity [2].

In the presence of early corneal opacities, the ophthalmologist has to consider a metabolic disease, and an early diagnosis improves the ocular and systemic prognosis. The cornea is a transparent tissue without vessels, and peripheral vascularization of the cornea can develop because of the chronic corneal oedema. Other alterations can be corneal exposure in relation to pseudo-exophthalmos that causes lacrymal film alteration and, at times, corneal ulcers.

#### Anterior segment

GAG deposition in the anterior segment can lead to increased intraocular pressure with consequent open-angle glaucoma (deposition in the trabeculocytes) and narrow-angle glaucoma (GAG in the peripheral cornea, iris and ciliary body) [8, 9]. Glaucoma has mostly been detected in patients with MPS I and MPS VI [8–12].

Most eyes with open-angle glaucoma show abnormalities on gonioscopic examination, especially in the trabecular meshwork [6]. Clinical signs of glaucoma include enlargement of the optic cup, ocular hypertension, and visual field defects and, if undetected, it progresses to irreversible blindness.

The decreased visual function occurs secondary to optic nerve damage. The earlier the onset of glaucoma, the poorer the visual prognosis becomes.

#### Retinal degeneration

GAG deposition within retinal pigment epithelial cells and in the photoreceptor matrix leads to progressive photoreceptor loss [13]. Optic nerve atrophy may also occur with advanced retinal degeneration [14]. Retinal degeneration clinically appears as sensitivity to light or night blindness [15]. It has a very insidious onset and is usually overshadowed by corneal opacities.

The initial funduscopic evaluation shows only arteriolar attenuation, while in late stages it shows marked pigment abnormalities and chorioretinal atrophy.

Peripheral visual constriction may gradually develop; clinically, it will be referred to as gradual tunnel vision and can be associated with clumsiness [4, 15]. Retinal degeneration and associated symptoms generally progress very slowly, with the time of onset depending on the severity of the phenotype [4]. Retinal degeneration is predominant in MPS III, and present sometimes in MPS I, II, and IV [3, 4, 15].

#### Optic disk swelling and optic nerve atrophy

GAG deposition causes thickening of the dura and sclera with consequent compression of the optic nerve resulting in optic disk swelling and subsequent optic nerve atrophy if compression is prolonged [3, 14]. Optic atrophy can also be attributed to direct GAG accumulation within ganglion cells. Another cause is terminal glaucoma damage.

#### Reduced vision

The correct development of visual function can be interrupted by reduced transparency of dioptric means, visual defects (astigmatism, hypermetropia), and strabismus [3, 4].

Storage of GAG in the cornea modifies the curvature, whereas in the sclera it reduces the axial length; these are the causes of hypermetropia in these patients.

Strabismus is common, and GAG infiltration of the extra-ocular muscles may also contribute. The esotropia is sometimes present as a consequence of raised intracranial pressure.

The acquired Brown syndrome (a rare form of vertical strabismus) is described in some cases due to mechanical limitation of movement of the superior oblique tendon [3, 4, 16].

### Ocular evaluation in MPS

An accurate evaluation of the eyes in patients with MPS may be challenging for several reasons. The ocular assessment should be tailored to the patient’s individual situation, taking into account the age, the presence of influencing factors such as severe photophobia, corneal clouding, or poor cooperation because of intellectual disability or behavioural disorders [17]. This last condition might require observation under anaesthesia.

We have also observed that it may be difficult to position the patients correctly on the evaluation device when they have skeletal abnormalities.

Recently, the Iris Camera (similar to the security tool used in airports) is used to allow the grading of corneal clouding; this examination shows high repeatability [18, 19].

#### Orthoptic evaluation and visual acuity measure

Evaluation of binocular function and ocular motility in children encompasses the assessment of muscle balance using cover test and/or prism/cover test [2]. Stereopsis (Lang I–II) is investigated. The visual acuity, if possible, is tested at distance and near views. We can use different tests according to the age: Teller acuity cards and Pesando pictures in preschool children, E test and ETDRS in school children. In all cases refraction after cycloplegia is recommended (cyclopentolate and/or tropicamide); severe corneal clouding or photophobia can make the examination difficult.

#### Slit-lamp examination

This examination permits the evaluation of corneal transparency, anterior chamber depth, pupil reaction, and lens transparency. Photographs are useful to evaluate the progression of corneal clouding. Corneal clouding may be initially asymptomatic or sometimes patients have photophobia. Generally, the clouding is diffuse from a mild haze to a milky ground glass appearance [4].

#### Intraocular pressure

The intraocular pressure can be falsely high because it is influenced by an increased central corneal thickness. The falsely high pressure is caused by rigidity of the cornea because of modified stromal architecture and GAG accumulations in the lysosomes [20]. Thickness can be measured with pachymetry or ocular coherence tomography (OCT). We also need to consider enlargement of the optic cup and visual field defects, and not just the intraocular pressure [12]. In poorly cooperative patients, an examination under anaesthesia should be planned for a correct and complete evaluation.

#### Fundus evaluation

This examination (dilated pupils) permits the evaluation of the optic disk (oedema, atrophy), retinal pigmentary changes, and retinal vessels modifications (reduced calibre) [2]. Corneal clouding can hinder visualization of the fundus leading to difficulties in diagnosing and monitoring changes in the retina and optic disc.

#### Visual field

Different techniques are used depending on that age and intellectual ability of the patients. Visual field changes are related to retinopathy, optic atrophy, and glaucoma. In non-cooperating children visual field tests cannot be performed. A useful assessment of significant defects can be made by simple comparison techniques; the examiner faces the child and attracts their attention centrally and then a target is introduced silently from the far periphery. A child with a normal visual field will make a very rapid head movement or a saccadic eye movement in the direction of the new stimulus [21].

#### Autorefraction

An autorefractor is a computerised device that permits objective refraction (to detect myopia, hypermetropia, and astigmatism) in cooperative patients. This objective instrument requires only that the patient remains still and fixes on a target within the instrument for few seconds. In earlier infancy, the traditional retinoscopy is the gold standard. This examination is strongly hampered in the presence of corneal clouding.

#### Optical coherence tomography (OCT)

**Fig. 2:**
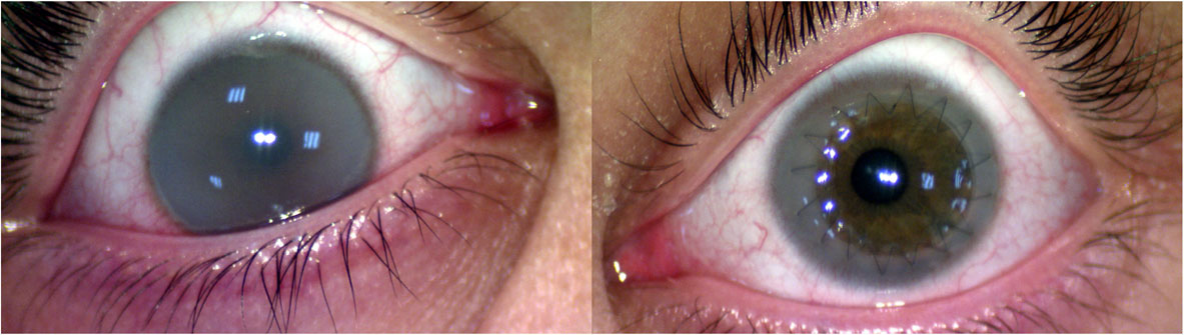
Corneal ocular coherence tomography (OCT). **a** Transparent cornea. **b** Corneal clouding in mucopolysaccharidosis (MPS) type I

It is useful to measure the corneal thickness and to study the layers of the cornea (Fig. 2). We can also analyse the central retina, the thickness of the retina, and findings of photoreceptor layers and optic nerve [13, 19]. The study of retinal nerve fibre layers (RNFL) is useful to analyse the optic nerve and changes in the follow-up of glaucoma.

#### Electrophysiological examinations

Visual evoked potentials can be useful for studying the visual pathways; swelling and atrophy of the optic nerve reduce the amplitude of the trace. Electroretinography is recommended in the case of suspicion of retinopathy [2, 22].

#### Ultrasounds

In all cases where corneal opacities can impede the funduscopic examination, echography can provide an evaluation of vitreous and retina [19]. The use of A-scan ultrasound is sometimes helpful to determine axial length. Ultrasound biomicroscopy (UBM) helps to examine the trabecular meshwork and the details of anterior segment structures.

#### Photography

Photography of the anterior segment and the fundus can be of particular value and provides documentation to assess changes over time [2]. Photographs are especially helpful to verify any improvement after medical or surgical therapy.

#### Examinations under sedation

Uncooperative patients need general sedation to perform a correct and complete ocular evaluation. The sedation permits the analysis of ocular features, including microscopic hand-held slit-lamp examination, funduscopic examination, pachymetry, tonometry, cycloplegic retinoscopy, ultrasound echography, and corneal and retinal imaging.

### Ocular management in MPS

#### Spectacle prescription

Refractive errors are common, especially hypermetropia and astigmatism [5]. In our patients we detected a huge incidence of these defects (Table 3). Lens prescription is difficult in these patients and requires experience and ability. We suggest using different modalities (autorefractometer, corneal topography, retinoscopic examination) to obtain the best correction. Photochromatic lenses are useful for reducing photophobia.

**Table 3:** Refraction defects in the patients with different types of mucopolysaccharidoses (MPS) evaluated in our department

MPS type	Hypermetropic astigmatism	Myopic astigmatism	Hyperopia	Myopia	Emmetropia
I (*n* = 17)	9	2	1	1	4
II (*n* = 14)	2	2	1	2	7
III (*n* = 6)		4			2
IV (*n* = 10)	6	2			2
VI (*n* = 4)	2		2		

Prescription spectacles, after examination with cycloplegia, are very important to improve the visual function [2, 23]. Despite this, sometimes these patients do not benefit from prescription spectacles. In the presence of amblyopia (lazy eye), orthoptic therapy with partial or total occlusion is performed to improve visual acuity.

#### Topical lubricants

After haematopoietic stem cell transplantation (HSCT), ocular complications are possible, particularly cataracts and dry eyes [24]. To improve keratoconjunctivitis sicca, we recommend topic treatment with lubricants. In severe cases, topical steroid and/or topical cyclosporine are used. Topical lubricants are beneficial in presence of pseudoexopthalmos for corneal exposure.

#### Antiglaucoma therapy

Topical beta-blocker therapy is the first choice when the intraocular pressure is high [2]. When medical therapy does not reduce the ocular pressure, glaucoma surgery should be considered.

### Aspects of corneal surgery in MPS

#### Corneal clouding: what is it and how does it manifest?

In cases of mucopolysaccharidosis (MPS), and common to all forms but more frequently in MPS IH (Hurler), MPS IH-S (Hurler Scheie), MPS VI (Maroteaux-Lamy), and MPS VII (Sly), the typical corneal manifestation consists of bilateral corneal clouding in the absence of an underlying flogistic condition; this is sometimes already present at birth but more frequently develops during the first years of life (Table 1) [25].

#### What are the most effective treatments for MPS?

The preferred therapy to regain transparency is corneal transplantation or keratoplasty, which becomes advisable when: 1) in very small children or patients that cannot undergo a visual acuity test the corneal opacity is so severe that it prevents the investigation of the other eye structures (lens and fundus); and 2) the corrected visus of patients goes below 0.4.

#### What specific diagnostic examinations are useful before transplant?

The following specific diagnostic examinations are useful: 1) slit lamp examination; 2) tonometry taking into consideration also the corneal pachymetry; 3) natural and corrected visus; and 4) corneal haze assessment with Pentacam densitometry programme [26].

Biomicroscopy is not useful because the opacity of the anterior corneal layers prevents the endothelial microscopy examination. When the possibility of having a presurgical visus is lacking for the abovementioned causes, it is strongly advised to perform an ERG (electroretinography), a visual evoked potential/visual evoked response (VEP/VER), and an echography to assess the if the retinal and optical nerve functionalities have already been compromised by the disease.

In some cases, due to the lack of cooperation by very young children or intellectually impaired patients, the above examinations must be performed under sedation or general anaesthesia; however, in these cases, especially in cases of heavily clouded corneas, the diagnostic advantage must be very carefully considered against the risks of anaesthesia, even more so if there is a presence of some form of airway abnormalities that are common in MPS patients.

#### Which transplant?

The preferred therapy has always been penetrating keratoplasty (PKP) but, more recently, many authors [27–29] have been favouring deep anterior lamellar keratoplasty (DALK) instead since, in this case, the patient’s own endothelium is retained thus preventing the possibility of endothelial rejection. This also seems to offer a better resistance to trauma later in life and, furthermore, it seems that the Descemet’s membrane (that in this case is retained) offers some resistance to the eventual re-accumulation of the GAG deposits that lead to an unavoidable reappearance of opaqueness of the transplanted graft.

Some authors [30] also suggest the possibility of performing a limbal stem cell transplantation to establish a barrier of healthy epithelial cells, further countering the return of the GAG deposits.

A very recent study by many authors [31] carried out a comprehensive review of keratoplasty in 32 patients from seven countries (Brazil, England, Finland, Germany, Portugal, Sweden, and USA; 16 bilateral and 16 monolateral) found that PKP was performed in 45 of the cases while DALK was only performed in three; at the last follow-up, successful results were reported in 63% of the first/only operated eye. Rejection episodes occurred in 23% of grafts. Ocular pathway comorbidities were identified in 63% of transplanted eyes; however, a clear graft was recorded at last follow-up in 94% (mean follow-up time 70 months, range 5–186 months).

#### Follow-up in the MPS patient who has undergone corneal transplantation

The purpose of systemic pharmacological treatment is delaying the return of the corneal haze which could happen as soon as the first year after transplant. Topical treatment should be carried out with a combination of antibiotics and steroids during the first month, followed by steroids and lubricants for at least 6 months. Check-ups should be executed twice during the first month, every 3 months during the first year, and twice a year afterwards until suture removal.

Lubricants must often be maintained, especially in the presence of keratoconjunctivitis sicca (dry eye syndrome) following HSCT or bone marrow transplantation (BMT).

During post-transplant follow-ups the following examinations should be performed if possible: 1) visus; 2) slit lamp examinations; 3) tonometry (to assess the presence of a concomitant glaucoma that would lead to transplant failure); 4) corneal topography; and 5) fundus examination (to evaluate an existing retinal or optic nerve degeneration). An optical correction prescription should not be carried out prior to refractive stabilization.

#### What are the possible complications for keratoplasty in MPS patients?

The intra- and post-surgical complications, other than those connected with the associated anaesthesiological risks, are no different from those connected to a common keratoplasty.

Parents of small children should be informed of the late complications that may occur, such as rejection, and it is important to evaluate any case of inflammation in the eye bulb represented by reddening and/or visual acuity loss in order to administer a topical therapy with steroids as early as possible.

#### Infection risk

The eye should be kept thoroughly clean, especially during the first month, and potentially dangerous activities should be prevented to avoid trauma that could lead to dehiscence.

The eventual clouding of the graft, in some cases occurring even within the first year from transplant, is not considered a complication since it is correlated with the presence of the MPS disease itself.

#### When should the suture be removed?

The suture should be removed no earlier than a year from transplant and following consideration of astigmatism and the state of the suture itself.

## Case report

During November 2016 an 11-year-old girl affected by MPS type VI had been referred to our system for medical examination. She had full and intense clouding in both eyes with a corrected visus of only 0.1 in the left eye and 0.2 in the right eye. She was therefore subjected to preliminary examinations (slit lamp, tonometry, and fundus), the execution of which proved very challenging; nevertheless, she did not present any major anomaly.

In December 2016 she underwent a penetrating keratoplasty procedure on the left eye. During the following check-ups the suture was fine, the graft was transparent, the intraocular pressure was normal, and the visus kept improving up to the value of 0.4–0.5 (corrected) measured during her latest check-up in March 2017. No inflammatory signs were detected and the patient was very satisfied with her new visual capability.

A corneal topography had been executed during every examination and showed, during the last check-up, a regular astigmatism of 3 D, and a biomicroscopy of the graft showed a cellular density of 2250 cells/mm^2^ and a graft pachymetry of 404 μm.

No subjective disorder has been reported by the extremely compliant little girl or by her parents.

The patient is following the above-mentioned postsurgical therapy protocol aimed at preventing inflammation and rejection.

**Fig. 3:**
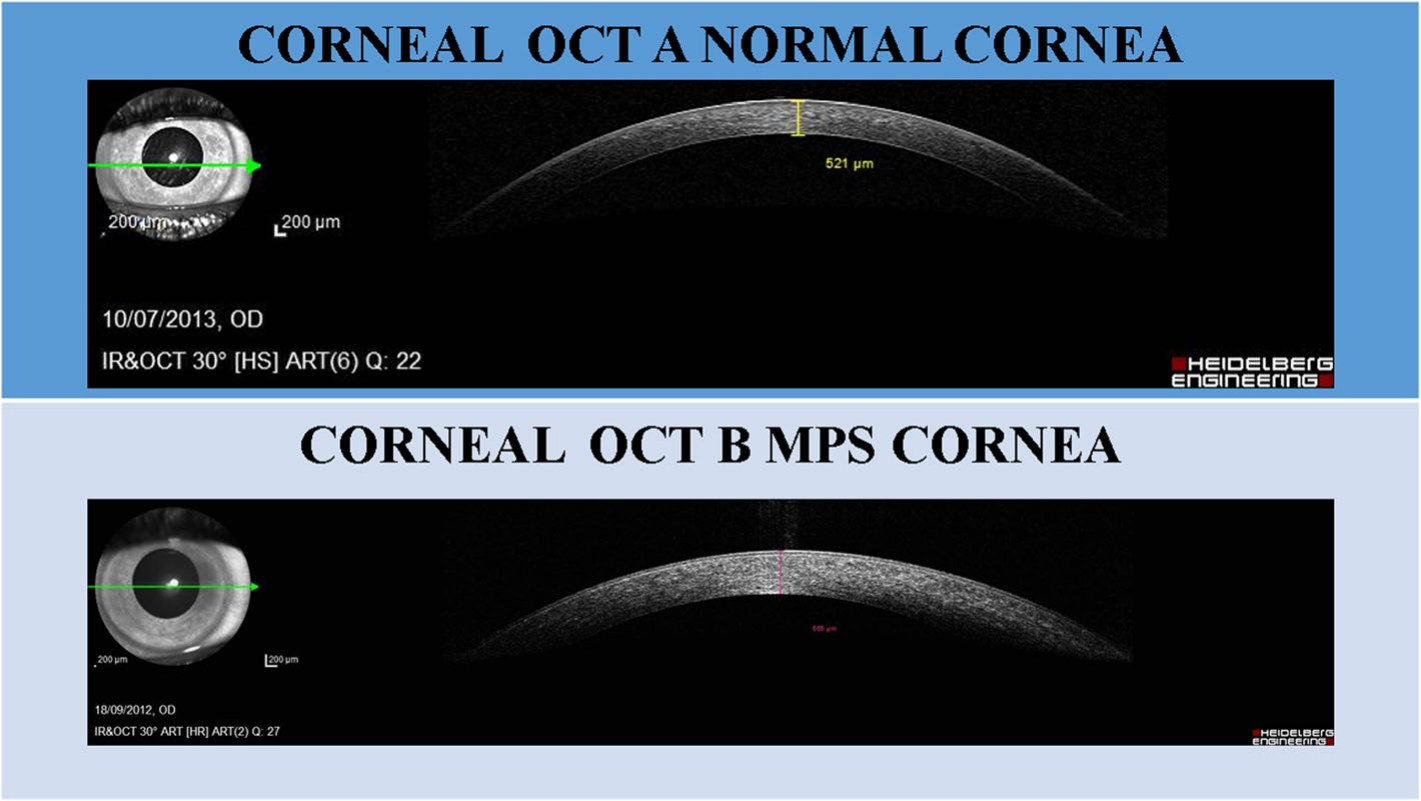
An 11-year-old MPS VI patient who underwent left corneal transplant. A transparent cornea with no inflammation in the left eye, and corneal clouding in the non-transplanted right eye

Given the good results already achieved and the high possibility of further improvement, especially when the suture will be fully settled allowing an optimal correction, a transplant will also be considered in the contralateral eye to regain a full binocular view and prevent, as far as possible, any phenomenon of amblyopia (lazy eye) (Fig. 3).

## Conclusions

Ocular manifestations are common in MPS [5]. A multidisciplinary approach is mandatory for MPS patients [6]. Annual ophthalmic evaluation is generally recommended, or more frequently in selective cases on request of the ophthalmologist [2]. Some patients with MPS and significant behavioural challenges will require examination under anaesthesia, and the anaesthesiological risk has to be considered. Early detection of ocular alterations is needed to improve the quality of life of these patients.

## Key messages

- Ocular manifestations are common in MPS
- Ocular examination is mandatory
- In selected cases, general sedation may be necessary

## Abbreviations

DALK: *Deep anterior lamellar keratoplasty*
ERT: Enzyme replacement therapy
GAG: Glycosaminoglycan
HSCT: Haematopoietic stem cell transplant
MPS: Mucopolysaccharidosis(e)s
OCT: Optical coherence tomography
PKP: Penetrating keratoplasty
